# Growth Traits and Sperm Proteomics Analyses of Myostatin Gene-Edited Chinese Yellow Cattle

**DOI:** 10.3390/life12050627

**Published:** 2022-04-23

**Authors:** Yuefang Zhao, Lei Yang, Guanghua Su, Zhuying Wei, Xuefei Liu, Lishuang Song, Chao Hai, Di Wu, Zhenting Hao, Yunxi Wu, Li Zhang, Chunling Bai, Guangpeng Li

**Affiliations:** State Key Laboratory of Reproductive Regulation and Breeding of Grassland Livestock, College of Life Science, Inner Mongolia University, Hohhot 010021, China; zhaoyf@imu.edu.cn (Y.Z.); mrknowall@126.com (L.Y.); suguanghua0707@163.com (G.S.); weizhuying2008@126.com (Z.W.); liuxuefei1006@126.com (X.L.); xiaoshuang2000@126.com (L.S.); h15248037201@163.com (C.H.); wudi2020imu@163.com (D.W.); ztinghao1996@126.com (Z.H.); yunxi_wu@sina.com (Y.W.); zhanglinmg@aliyun.com (L.Z.)

**Keywords:** gene editing cattle, MSTN, CRISPR/Cas9, sperm, FLQ proteomic

## Abstract

Chinese Yellow Cattle, an ancient and domesticated breed for draft service, provide unique animal genetic resources with excellent genetic features, including crude feed tolerance, good stress resistance, strong adaptability, and tender meat quality; however, their production performance and meat yield are significantly inferior. Herein, the myostatin gene (*MSTN*), a negative regulator of skeletal muscle development, was knocked out by CRISPR/Cas9 technology. Eight *MSTN* gene-edited bull calves (MT) were born, and six of them are well-developed. Compared with the control cattle (WT), the growth trait indexes of MT cattle were generally increased, and the hindquarters especially were significantly improved. The biochemical indexes and the semen characteristics demonstrated that MT bulls were healthy and fertile. Consistent with our conjecture, the wobble and beating of MT bull spermatozoa were significantly higher than that of WT. Nine sperm motility-related proteins and nineteen mitochondrial-related proteins were identified by up-regulation in MT bull spermatozoa using FLQ proteomic technique and act to govern sperm flagellum assembly, organization, and beating and provide sufficient energy for sperm motility. The current study confirmed that the *MSTN* gene-edited Chinese Yellow cattle have improved growth traits and normal fertility, which can be used for beef cattle production and breeding.

## 1. Introduction

To date, at least six functional mutations have been found in the myostatin (*MSTN*) gene of different breeds of cattle, including Belgian Blue, Piedmontese, Charolais, Blonde d’Aquitaine, Limousin, and German Gelbvieh. The natural deletion of 11 bases at nt821-831 of *MSTN* gene in Belgian Blue cattle results in the loss of three amino acids at 275, 276, and 277; the reading frame also shifts from 274, and the stop codon advances to 287 [1]. A single-base mutation of G to A occurs at nt938bp of the third exon region of *MSTN* gene in Piermont cattle, resulting in the mutation of Cys to Tyr at position 313 (C313Y) of the amino acid sequence, which occurs at the fifth cysteine of the nine cysteines in the highly conserved TGF-β superfamily [1]. In Charolais cattle, the mutation from C to T at nt238bp in the second exon of MSTN results in the mutation from Glu to X (stop codon) at the 204th position [2]. A single-base mutation of C to A occurs in the first exon nt282bp of MSTN in Limousin cattle, which results in the mutation of amino acid sequence from Phe to Leu at position 94 [3]. In Blonde d’Aquitaine, a T to G mutation occurs at nt1234bp of the second intron of *MSTN* gene, resulting in 41 bp increase in front of the third exon and an amino acid sequence shift mutation and termination, which result in total loss of the amino acid sequence encoded by the third exon [4]. The mutation of a single base from T to C at nt191bp of the first exon of MSTN in German Gelbvieh results in the mutation of the 64th amino acid sequence from Leu to Pro, and the protein structure is thus changed [5]. These mutations in *MSTN* genes occur in different breeds of cattle and have all changed the structure of MSTN secreted proteins, resulting in the inactivation of MSTN proteins, relieving the inhibition of MSTN on muscle development, and then inducing a muscular double-muscle phenotype.

Compared with non-mutant cattle, the growth rate and muscle yield of MSTN mutant cattle are significantly increased, the fat content of beef significantly decreased, and the lean meat significantly increased compared to that of normal cattle. With the same 600 kg of live-weight, the double-muscle Belgian Blue cattle have 70% the slaughter rate compared to the 64% of normal cattle, and the cold-carcass weights are 420 kg and 384 kg, respectively, which indicate the slaughter rate and net meat rate are significantly improved in double-muscle cattle [6].

Casas et al. analyzed the production performance of 536 Belgian Blue cattle hybrid offspring; the slaughter weight was significantly higher than that of normal cattle (*p* < 0.05), and the hind leg and rump weight were significantly higher than that of normal cattle (*p* < 0.001). The weight of tenderloin meat was significantly increased (*p* < 0.001), and the area of eye muscle was significantly increased (*p* < 0.05) [7,8]. These results indicate that the meat yield and meat quality of Belgian Blue cattle heterozygous progenies are significantly better than that of normal beef cattle. Genotypes and number of alleles have significant effects on muscle grade, fat thickness, and production difficulty. In both Belgian Blue and Piermont mutants, heterozygous progenies are characterized by high lean meat and carcass weight. As a result, hybrid cattle can be maximized during production breeding to improve carcass output. Wheeler et al. analyzed tenderness of four kinds of muscle tissues, including longissimus dorsi, gluteus medius, semimembranosus, and biceps femoris of the double-muscled Piermont cattle, and found that the tenderness and connective tissue quantity of the heterozygous and homozygous mutant cattle are significantly higher than those of wild-type cattle, and the juiciness of all muscle types in homozygous mutants is less than that in heterozygous and wild types [9]. Three species of bulls, namely Piermont, Limousin, and Hereford, were bred with cows to produce F1- and F2-generation cattle. Short et al. showed that the birth weight of F2 offspring of double-muscled Piermont cattle was significantly higher than that of the offspring of Hereford cattle, the meat production performance was significantly improved, and the fat content was significantly reduced. The birth weight of the offspring of Limousin cattle was lower than Piermont but higher than the Hereford [10]. Wiener et al. studied the growth, body condition, and meat production performance of the crossbreed of Belgian Blue bulls and South Devon cows. The heterozygous hybrid cattle had significantly improved meat yield, reduced fat thickness, and improved muscle grade but increased calving difficulty [11]. Casas et al. reported that the hybrid cattle crossed by Belgian Blue bulls with different breed cows had significantly higher birth weight, weaning weight, and slaughter weight and lower fat content than those of non-mutant calves [12,13,14]. Sellick et al. found that Limousin cattle with the F94L mutation had enhanced muscle development, which could increase leg muscle by 5.5% and eye muscle area by 2.3% [15]. Allais et al. studied two kinds of French *MSTN* genes with Q204X and nt821 mutations in Charolais, Limousin, and Blonde d’Aquitaine cattle and found that the carcass fat content, intermuscular fat content, and collagen content of the calves with Q204X mutation were significantly reduced compared with the non-mutated cattle [16].

Historically, Chinese indigenous cattle breeds have been selected for draft service, with speed and endurance as the breeding criteria, and the cattle are generally strong in the front, with strong limbs but an underdeveloped body and streamlined hips. Compared with Angus, Simmental, Hereford, Limousin, or Charolais beef cattle breeds, the meat performance and production performance of Chinese indigenous cattle breeds are significantly inferior. However, the old indigenous breeds of Chinese cattle contain extremely rich and excellent genetic germplasm resources and have the advantages of rough feeding tolerance, good stress resistance, strong adaptability, and tender meat. In the past 10 years, in order to increase the growth and meat performance of Chinese indigenous cattle, we have targeted the myostatin gene by CRISPR/Cas9 gene-editing technology, produced artificial double-muscle bulls, and bred Chinese indigenous cattle into a new beef cattle breed with high yield and quality. According to the literature, the fetuses of Belgian Blue cattle with myostatin mutation in exon3 developed too fast and had excessive birth weight, resulting in a high rate of dystocia, especially for the homozygous [12]. The dystocia rate of Limousin with myostatin mutation in exon1 was significantly lower than that of Piedmont and Simmental cattle, and the survival rate of early Limousin calves was higher [13,14]. Therefore, the first exon of myostatin gene was selected as the target of gene editing in this study. The breeding of gene-edited cattle is realized through artificial insemination with their semen. Sperm quality is a key factor in determining pregnancy rates and maintaining production, especially for cryopreserved semen. Therefore, the sperm quality of gene-edited, cloned cattle were analyzed in this studied.

## 2. Materials and Methods

### 2.1. Ethics Statement

All experimental procedures in this study were consistent with the National Research Council Guide for the Care and Use of Laboratory Animals. All protocols were approved by the Institutional Animal Care and Use Committee at Inner Mongolia University (Approval: SYXK 2014-0002). Chemicals were purchased from Sigma Chemical Co. (St. Louis, MO, USA) unless otherwise indicated. Primers were synthesized by Takara Biotechnology Dalian Co., Ltd. (Dalian, China).

### 2.2. Construction of CRISPR/Cas9 Vectors

To generate vectors expressing Cas9 and sgRNA for targeting MSTN exon 1, three sgRNAs, named sgRNA-1, sgRNA-2, and sgRNA-3, to target bovine *MSTN* gene were designed using online software (http://crispr.mit.edu (accessed on 20 May 2016)) in cattle. CRISPR/Cas9 compound plasmid was provided by Nanjing YaoShunYu Biological Technology Co., Ltd., (Nanjing, China). The sgRNAs sequences were as following: Guide #1: GCAGGACTACTCACACTCCGTGG, RC: CCACGGAGTGTGAGTAGTCCTGC; Guide #2: CTACTCACACTCCGTGGGCATGG, RC: CCATGCCCACGGAGTGTGAGTAG; Guide #3: CGGAGTGTGAGTAGTCCTGCTGG, RC: CCAGCAGGACTACTCACACTCCG.

In order to improve the targeting efficiency, three sgRNAs were connected in a series in the same carrier. First, a single sgRNA was constructed into the pYSY-TA clone vector and then into the final cutting vector as independent sgRNA-expression vector. Three target plasmids, namely pYSY-TA-U6-sgRNA 1–3, were confirmed as successfully constructed by sequence alignment.

### 2.3. Transfection of Bovine Fetal Fibroblast Cells with CRISPR/Cas9 Cutting Vector and Screening of Positive Gene-Edited Cells

The bovine fetal fibroblasts (BFFs) were prepared and cultured as follows. Chinese yellow cattle (Luxi Yellow cattle) male fetuses were obtained via hysterectomy of a pregnant (day 45) cow and washed in Dulbecco PBS (DPBS) (Gibco, Grand Island, NY, USA). Heads, tails, limbs, and viscera were removed, and the remaining tissues were washed again in DPBS and cut into cubes. Next, these tissues cubes were digested in cell culture medium containing 20% fetal bovine serum (FBS) (Gibco, Grand Island, NY, USA), 200 U/mL collagenase IV (type IV, 260 U/mg, Gibco), 25 U/mL DNase I (2000 U/mg, Beyotime, Haimen, China), and 1% penicillin/streptomycin (Gibco) and cultured for 4–6 h at 37 °C under 5% CO_2_ in humidified air. Isolated BFFs were digested from the culture dishes using 0.05% (*w*/*v*) trypsin in DPBS and centrifuged at 500× *g* for 5 min. After removal of the supernatant, BFFs were resuspended and cultured in new 10 cm cell culture dishes. Then, the BFFs were frozen in FBS containing 10% dimethylsulfoxide. In the following experiments, cells were thawed and cultured in DMEM/F12 with 10% FBS at 37 °C under 5% CO_2_ in humidified air.

The correctly sequenced monoclonal bacterial solution was resuscitated and placed under extended culture, and the corresponding pSpCas9-sgRNA cleavage plasmid was extracted with endo-free Plasmid Mini Kit II. The BFFs were transfected according to the Lipofectamine™ 3000 Transfection Reagent (Thermo, Waltham, MA, USA) instructions, and pSpCas9 without sgRNA was used as the control group. After 72h culture of the transfected cells, the genomic DNA was extracted by DNeasy Blood and Tissue Kit, and the cutting efficiency was detected by T7EI nuclease.

After the donor template and donor vector were co-transfected with fetal fibroblasts, single cells were sorted into 96-well plates by flow cytometry for monoclonal culture. After 10 to 15 days of culture and two passages, when the proliferation of monoclonal cells reached 10^5^ cells, 1/3 of the cells were extracted for genome extraction and PCR detection for site-directed mutation, and the remaining cells were frozen for future use. The confirmed positive gene-edited monoclonal cells were cultured and used as the positive clonal donor cell of SCNT (somatic cell nuclear transfer) to produce cloned bulls.

### 2.4. Production of MSTN Gene-Edited Bulls by SCNT

The cloned bulls were produced by SCNT, and the experimental procedures were carried out in accordance with our published articles [17,18]. Briefly, oocytes of Luxi Yellow cattle with the first polar body were selected for enucleating after maturation in vitro for 20 h. The polar body was removed, and a positive clonal donor cell was injected into the peri-vitelline space of the enucleated oocyte. Nuclear-cytoplast couplets were fused by electro-fusion. The fused embryos were activated and cultured to blastocysts. Seven-day SCNT blastocysts were non-surgically transferred (one or two embryos per recipient) into the uterine horn ipsilateral of synchronized Simmental recipients at day 7 after estrus. Surrogate Simmental cows were 2 to 3 years old under the same pasture management and feeding environment. Pregnancy was diagnosed by rectal palpation or ultrasonography at 60 days after blastocyst transfer (day 0). The cloned calves were delivered naturally or by manually assisted delivery. The birth weight was measured immediately after birth. Genomic DNA was extracted from the blood of cloned calves for genotyping PCR. The primers used in PCR amplification were the same as positive gene-edited cells detection.

### 2.5. Growth Parameters Detection

The *MSTN* gene-edited Luxi Yellow cattle (named MT cattle) and control group without gene editing, the wild-type Luxi Yellow cattle (named WT cattle), were raised under the same conditions. The feed plan of the bulls was made according to feeding standard of beef cattle as outlined in Chinese NY/T815–2004. The feeding of beef cattle is divided into four main stages: (1) Newborn to 2 months of age: Calf is mainly fed with breast milk. After 1 month of age, a total of 0.25–0.40 kg commercially manufactured special feed for calves is fed by dividing into multiple times. The amounts of Leymus chinensis and warm water are based on voluntary intake. (2) Three months of age to weaning (about four months of age): Calf is fed with breast milk and a total of 0.50–0.75 kg commercially manufactured special feed for calves by adding multiple times. The amounts of Leymus chinensis and warm water are based on voluntary intake. (3) Weaning to 12 months of age: Bull is fed with 1.50–2.50 kg commercially manufactured special feed for bulls and 5–6 kg roughage, dividing two times. The amounts of warm water are based on voluntary intake. (4) Finally, at 13–24 months of age: Bull is fed with 2.00–3.00 kg commercially manufactured special feed for bulls and 7–10 kg roughage, dividing two times. The amounts of warm water are based on voluntary intake.

The commercially manufactured special feed for calves and bulls were purchased from Inner Mongolia Mengyuankang Feed Co., Ltd., (Hohhot, China). The main materials of commercially manufactured beef feed include corn, soybean meal, cottonseed, yeast feed, distillers dried grains with solubles, limestone, trace minerals, vitamins premix, sodium chloride, etc., which was composed of total mixed ration as shown in Table 1. The roughage is made of corn straw (30%), alfalfa (20%), and ensilage (50%) by mixing, crushing, and stirring.

The growth parameters were recorded at month 0 (newborn), 3, 6, 9, 12, 15, 18, and 24, respectively. The detected growth parameters included body weight (BW, kg), body length (BL, cm), shoulder width (SW, cm), cross-hip width (CW, cm), hip width (HW, cm), body height (BH, cm), cross-hip height (CH, cm), hip height (HH, cm), chest girth (CG, cm), and abdominal girth (AG, cm).

### 2.6. Blood Biochemical Analysis of Cattle

Blood samples were collected from jugular veins of both MT and WT cattle at months 9, 12, 15, and 18, respectively, to prepare serum samples. The routine blood tests and physiological and biochemical indexes were measured using a blood biochemical analyzer (Cobas C311, Roche) as described in our most recent report [19]. The blood physiological and biochemical analyses included glucose (GLU), aspartate aminotransferase (AST), alanine aminotransferase (ALT), total protein (TP), albumin (ALB), creatine kinase (CK), high-density lipoprotein cholesterol (HDL-C), low-density lipoprotein cholesterol (LDL-C), amylase activity (PAMY), creatinine (CREA), lactate dehydrogenase (LDH), actual bicarbonate (AB), cholinesterase (CHE), cholesterol (TC), lactic acid (LA), triglyceride (TG), urea (BUN), and lipase (LIP). The indexes of routine blood tests were lymphocyte ratio (LYM%), lymphocyte count (LYM), neutrophil count (NEUT), red blood cell (RBC), hematocrit (MCV), platelet count/blood platelet count (PLT), plateletocrit (PCT), mean platelet volume (MPV), basophil (BA), basophil ratio (BA%), allergy lymphocyte ratio (AL%), allergy lymphocyte (AL), and hemoglobin (HGB).

### 2.7. Semen and Sperm Analysis

The collection and detection of bovine semen were carried out as described in our recent report [20]. Briefly, semen was collected by pseudo-vagina method twice a week. Two jumps were asked per collection, and the semen was pooled for analysis and cryopreservation. Semen density was measured by a sperm density analyzer (IMV Technologies China, Shanghai, China, Product No. 014475). Appropriate diluent solution (OptiXcell, 026218, IMV Technologies China) was added according to semen density so that each straw (0.25 mL, 00567, IMV Technologies China) contained 2 × 10^7^ spermatozoa. The semen was then sealed into straws by an MRS1 single-head potting machine (IMV Technologies China, Product No. 022989). After 2–4 h balance, the semen was gradually frozen using a digitcool programmed automatic cryometer (IMV Technologies China, Product No. 007263) and frozen in liquid nitrogen for cryopreservation. An SCA automatic semen analyzer (IMV Technologies China, Product No. 024905) was used to analyze sperm motility, the velocity average line (VCL, mm/s), the velocity straight line (VSL, mm/s), the velocity average pathway (VAP, mm/s), the linearity coefficient (LIN, %), the straightness coefficient (STR, %), the wobbling (WOB, %), and the beta cross-frequency (BCF, Hz).

The sperm and seminal plasma samples were separated by centrifugation at 1000× *g* for 10 min. The supernatant was seminal plasma, and the sperm was washed with phosphate-buffered saline (PBS) three times. ICP-MS was used to determine the element concentration of seminal plasma, including Na, K, Ca, Se, Hg, Cu, Zn, Mg, Al, Fe, Mn, Pb, Ni, Cr, Sb, Ba, Sn, Sr, Co, V, and Ti. The sperm plasma membrane integrity was measured by the hypoosmotic swelling test (host). The sperm acrosome integrity was assessed by Coomassie brilliant blue staining. Sperm mitochondrial sheath integrity was tested by staining with Rhodamine-123 and PI and analyzed by flow cytometer.

### 2.8. Sperm Protein Extraction and Digestion

The sperm protein extraction and digestion were carried out as described in our most recent report [21]. Sperm sample was lysed by ultra-sonication. After centrifugation, the supernatant was collected as the sperm protein sample, and its concentration was determined by using the BCA Protein Assay Reagent Kit (23225, Thermo). The sperm protein sample (1 mg) was reduced, alkylated, and digested, respectively, to obtain the peptide sample, which was desalted using a C18 desalting column (Oasis HLB 1cc 30 mg Extraction Cartridges, Waters, Milford, MA, USA).

### 2.9. LC-MS/MS Analysis

An Orbitrap Fusion mass spectrometer (Thermo-Fisher Scientific, Waltham, MA, USA) and an EASY nLC1000 system (Thermo-Fisher Scientific, Waltham, MA, USA) were used to analyze the fractionated peptides. The Orbitrap Fusion was interfaced with the Nanospray Flex Ion Source. Each peptide sample was injected onto a PepMap C18 RP nano trap column (Acclaim PepMapTM 100, C18, 75 μm × 2 cm, 3 μm, 100Å, nanoViper, Thermo) with nanoViper Fittings at 20 μL/min flow rate and separated on a PepMap C18 RP nano column (Acclaim PepMapTMRSLC, C18, 75 μm × 25 cm, 2 μm, 100Å, nanoViper, Thermo). Reverse-phase separation of peptides was performed using buffer A (0.1% formic acid in water) and buffer B (0.1% formic acid in acetonitrile) with 300 nL/min flow rate, under a 100 min gradient (2–13% buffer B for 27 min, 13–32% buffer B for 65 min, 32–40% buffer B for 5 min, 40–90% buffer B for 1 min, 90% buffer B for 2 min). The eluted peptides were analyzed on the Orbitrap Fusion, which was operated in the positive ion mode with a spray voltage set at 2.3 kV and the source temperature at 275 °C. The instrument was operated in the data-dependent acquisition (DDA) mode using an FT mass analyzer for one survey MS scan on selecting precursor ions, followed by Top 3 s data-dependent HCD-MS/MS scans for precursor peptides with 2–7 charged ions above a threshold ion count of 20,000 with normalized collision energy of 32%. For these experiments, the MS survey scans were acquired over a mass range of *m*/*z* 350–1550 with the Orbitrap analyzer at a resolving power of 120,000, and MS/MS scans were acquired using an Orbitrap with a resolution of 30,000. Dynamic exclusion duration was 45 s. All data were acquired with Xcalibur 4.1 operating software and Orbitrap Fusion Tune 3.0.2041 (Thermo-Fisher Scientific, Waltham, MA, USA).

All MS/MS raw data were processed and searched using the Sequest HT algorithm within the Proteome Discoverer 2.4 (PD 2.4, Thermo-Fisher Scientific, Waltham, MA, USA). The Bos Taurus RefSeq sequence database with 41,062 entries downloaded from the NCBI database (13 October 2018) was used for database search. Identified peptides were filtered for maximum 1% FDR using the Percolator algorithm in PD 2.4 along with additional peptide confidence set to high. The mass tolerance for precursor ions was set to 20 ppm. Enzyme specificity was set to full cleavage by trypsin, and two maximum missed cleavage sites were permitted. The carbamidomethyl of cysteine was set as the fixed modification, and variable modifications included oxidation of methionine.

### 2.10. Bioinformatics Analysis

DAVID bioinformatics resource (https://david.ncifcrf.gov; version 6.8, 23 October 2021) was used for Gene Ontology (GO) analysis and domain annotation. The regulatory pathways were analyzed using Kyoto Encyclopedia of Genes and Genomes (KEGG) (https://www.genome.jp/kegg/, 23 October 2021). The STRING database (https://string-db.org, version 11.5, 25 October 2021) was used to obtain the protein interaction relationships.

### 2.11. Statistical Analysis

Quantitative experiments consisted of at least three independent replicates. The data are shown as mean ± standard deviation (SD). Data were statistically analyzed using GraphPad Prism software (*t*-test), and a *p*-value < 0.05 was statistically significant and marked as *. *p*-Value < 0.01 marked as **.

## 3. Results

### 3.1. Generation and Identification of MSTN Gene-Edited Chinese Yellow Cattle

**Figure 1:**
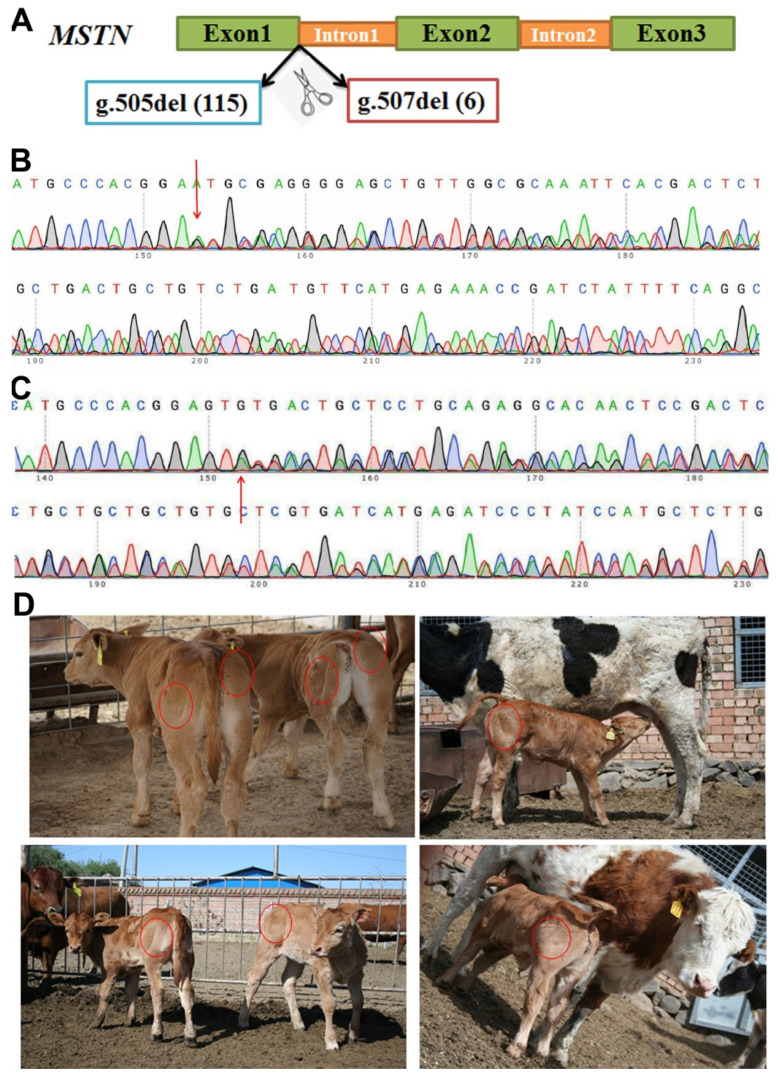
Generation and identification of *MSTN* gene-edited Chinese Yellow cattle. (**A**) The schematic diagram of *MSTN* gene and the editing sites; (**B**) sequencing chromas for g.507del (6) in the *MSTN* gene; (**C**) sequencing chromas for g.505del (115) in the *MSTN* gene (**D**). Photographs of *MSTN* gene-edited calves.

Two kinds of positive gene-edited cell lines were used as donor (Figure 1A), and 58 and 62 gene-edited embryos were obtained by somatic cell nuclear transfer, respectively. Total of 62 recipient cows were selected, and each cow had 1–2 embryos transferred into the uterine horns. The remaining cloned blastocysts were cryopreserved. Pregnancy tests were performed around 60 days after embryo transfer; nine of the recipient cows were pregnant, with a pregnancy rate of 17.3%. Eight gene-edited bull calves were successfully born, with the average gestation period was 289 days; the shortest gestation period was 276 days (one cow), and the longest was 295 days (one cow). The average birth weight was 33.6 kg, the smallest was 25.5 kg, and the largest was 43.5 kg. Unfortunately, two calves were weak and died at 45 and 110 days after birth, respectively. The other six calves were developed normally and named MT1, MT2, MT3, MT4, MT5, and MT6, respectively (Figure 1D). The genomic DNA was extracted from the calves to identify the DNA sequence of *MSTN* gene by PCR. The results of sequence analysis were consistent with the expectation that 6 bp (g.507 Del (6)) was deleted at 507 site (Figure 1B), or 115 bp (g.505 Del (115)) was deleted at 505 site (Figure 1C).

The growth traits and blood physiological and biochemical indexes of the six MT bulls from birth to 24 months of age were measured systematically, and semen were collected and analyzed after 18 months old.

### 3.2. Analysis of Growth Traits of MSTN Gene-Edited Cattle

#### 3.2.1. Body Weight of *MSTN* Gene-Edited Cattle

The body weights were traced, measured, and analyzed from birth to 24 months of age for *MSTN* gene-edited cattle (MT) and wild-type control cattle (WT). The results showed that the birth weight yielded no significant difference between MT cattle and WT cattle (Table 2). However, after 3 months old, the body weight of MT cattle was 111.4 kg, while that of WT cattle was 99.0 kg, which showed a significant difference. With increasing month age, the weight gain rate of MT cattle was significantly higher than that of WT cattle. The increase rates reached 10.2% and 16.8% at 12 and 24 months, respectively. At 24 months of age, the body weight of MT cattle was 593.59 kg, while that of WT cattle was 485.73 kg, which showed a significant difference (*p* < 0.05).

#### 3.2.2. Body Size of *MSTN* Gene-Edited Cattle

**Figure 2:**
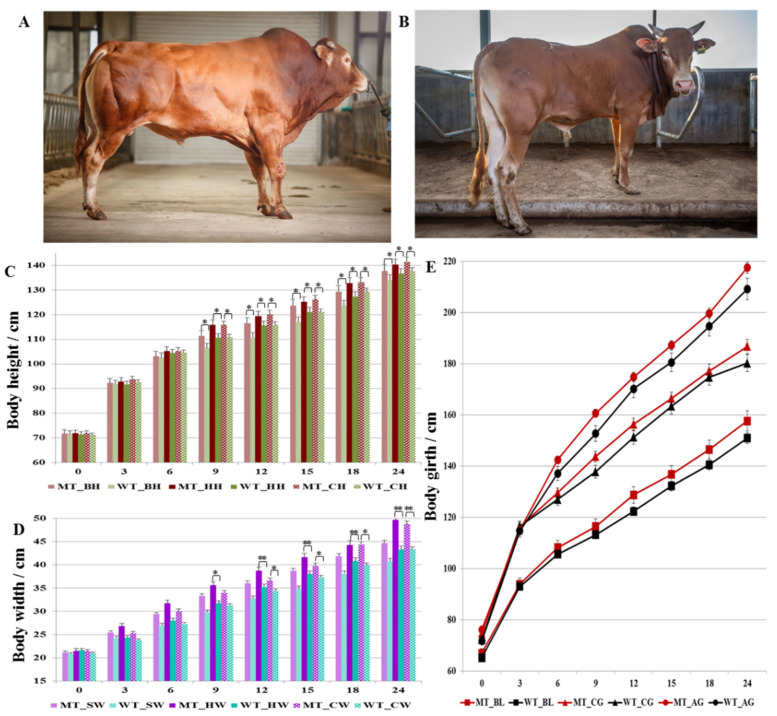
Growth traits during the growth of MSTN-edited cattle. (**A**). *MSTN* gene-edited cattle (MT); (**B**) wild-type control group (WT). (**C**) Analysis results of bovine body height (BH), cross-hip height (CH), and hip height (HH). (**D**) Analysis results of bovine shoulder width (SW), cross-hip width (CW), and hip width (HW). (**E**) Analysis results of bovine body length (BL), chest girth (CG), and abdominal girth (AG). * stands for *p* < 0.05, ** stands for *p* < 0.01.

Body height (body height, hip height, and cross height), body width (shoulder width, hip width, and cross width), body oblique length, chest girth, and abdominal girth were measured and compared between MT cattle and WT cattle from birth to 24 months of age, respectively. The results (Figure 2) showed that there were no significant differences in body height, hip height, and cross height between the two groups before 6 months age although the MT cattle were slightly higher. From 9 month of age, the body height, hip height, and cross height of MT cattle were significantly higher than that of WT cattle (Figure 2C).

With increasing month age, the width of MT cattle increased faster than that of WT, but there was no significant difference in shoulder width by *t*-test; however, hip width and cross width showed significant difference after 9 months of age, and the difference was increased (Figure 2D). The *p*-values of cross width between MT and WT cattle were 0.03, 0.02, 0.03, and <0.01 for 12, 15, 18, and 24 months of age, respectively. The *p*-values of hip width between MT and WT cattle were 0.0252, 0.0078, 0.0001, 0.0003, and <0.0001 for 9, 12, 15, 18, and 24 months of age, respectively. The changes of hindquarters width were more than that of the body height for the *MSTN* gene-edited cattle, which indicated that the weight gain of MT cattle was mainly achieved by the increased width of hindquarters.

Compared the body oblique length, both chest girth and abdominal girth showed no significant difference between MT and WT cattle (Figure 2E). However, the difference of abdominal girth was larger than that of chest girth between MT and WT cattle, which further implied that the hindquarters were greatly increased in MT cattle. The chest girth and abdominal girth of MT cattle began to differ from WT cattle after 6 months of age, but the difference was not significant. *MSTN* gene editing did not change the length of the cattle; thus, it had little effect on bone development.

### 3.3. The Physiological and Biochemical Indexes of MSTN Gene-Edited Cattle

Blood samples were collected from both MT and WT cattle jugular veins at 9, 12, 15, and 18 months of age, respectively, for the routine blood tests and physiological and biochemical indexes measurement. The results of routine blood test showed that there was no significant difference between MT and WT cattle (Table 3). In terms of serum physiological and biochemical indexes (Table 4), the high-density lipoprotein (HDL), low-density lipoprotein (LDL), alpha-amylase (PAMY), lactic acid (LA), urea (BUN), and lipase (LIP) were significantly increased in MT cattle compared with WT cattle, while the content of glucose (GLU) was significantly decreased in MT cattle.

### 3.4. Semen Characteristics of MSTN Gene Edited Cattle

Semen volume, sperm density, fresh sperm motility, and frozen sperm motility of MT and WT bulls were analyzed (Table 5). The average semen volume was 4.8 ± 0.7 mL Vs. 5.6 ± 0.3 mL, sperm density was 1252 ± 180 × 10^6^/mL Vs. 1686 ± 306 × 10^6^/mL, fresh sperm motility was 73.9 ± 7.2% Vs. 78.3 ± 3.0%, and motility of frozen-thawed sperm was 40.8 ± 14.5% Vs. 55.8 ± 8.1% between MT cattle and WT cattle, respectively, which showed no significant difference. The individual differences of freezing resistance of sperm from different bulls were significant. The average frozen sperm motility of MT3 was 62.1 ± 8.8%, while that of MT4 was only 18.8 ± 11.6% with instability.

According to the results of elements analysis (Table 6), the concentrations of Na, K, Ca, Mg, Fe, and Zn in bovine seminal plasma were higher than 1 mg/mL, and the concentrations of Mn, Ni, Pb, Sn, Sb, and Co were lower than 50 μg/mL. Compared with WT cattle, the contents of K, Hg, and Sr in MT cattle were up-regulated, while the contents of V, Cu, Mn, Sn, Sb, and Co were down-regulated.

### 3.5. Characteristics of Cryopreserved Spermatozoa of MSTN Gene-Edited Cattle

The sperm motility-related parameters of cryopreserved spermatozoa were detected by SCA automatic semen analyzer, including VCL (curvilinear velocity), VSL (velocity straight line), VAP (velocity average pathway), LIN (linearity coefficient), STR (straightness coefficient), WOB (wobble), ALH (amplitude of lateral head displacement), and BCF (beat cross-frequency). The results showed that WOB and BCF of MT cattle spermatozoa were significantly higher than that of WT cattle (*p* < 0.05) (Table 7).

**Figure 3:**
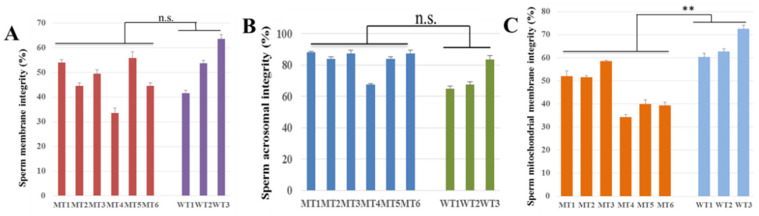
Results of sperm substructural integrity. (**A**) Plasma membrane integrity; (**B**) acrosomal integrity; and (**C**) mitochondrial membrane integrity. n.s., not significant difference, ** stands for *p* < 0.01.

Meanwhile, the plasma membrane integrity, acrosome integrity, and mitochondrial membrane integrity of the cryopreserved spermatozoa were also examined. The plasma membrane integrity was measured by hypotonic swelling method, and the results showed that the plasma membrane integrity of MT cattle sperm was 47.0 ± 8.1%, and MT4, with the lowest measurements, was only 33.5% (Figure 3A). The plasma membrane integrity of WT cattle sperm was 53.0 ± 11.0%, which was not a significant difference compared to MT cattle (*p* > 0.05). The acrosome integrity of sperm is an important index to evaluate sperm quality, and no significant difference between MT group (83.1 ± 7.9%) and WT group (72.0 ± 10.1%) (*p* > 0.05) was observed (Figure 3B). Sperm mitochondria are easily damaged during semen freezing and thawing, and thus, the sperm mitochondrial membrane integrity was analyzed using flow cytometer. The results showed that the mitochondrial membrane integrity of MT1, MT2, and MT3 were higher than 50%, while that of MT4, MT5, and MT6 were lower than 40%. The mitochondrial membrane integrity of WT cattle was higher than 60%, which was significantly higher than MT cattle (*p* < 0.05) (Figure 3C).

### 3.6. Proteomic Analysis of Cryopreserved Spermatozoa of MSTN Gene-Edited Cattle

**Figure 4:**
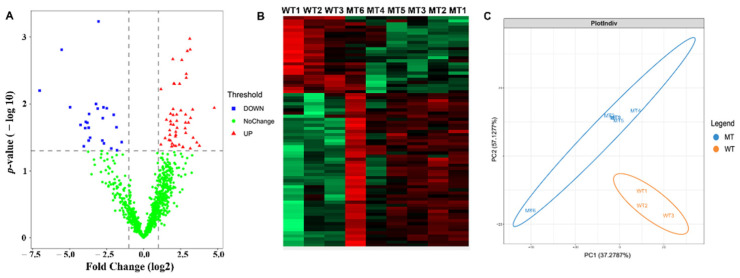
Results of bovine sperm proteome. (**A**) Volcano plot; (**B**) hierarchical cluster analysis plot; and (**C**) principal component analysis plot.

The cryopreserved sperm proteins of six MT cattle and three WT cattle were analyzed by free-label quantitative proteomics. A total of 2279 proteins were identified in nine bovine sperm samples, of which 1009 proteins had quantitative values in all nine samples. The screening conditions for proteins with quantitative values were Razor + Unique Peptides at least equal to 2. In total, 75 differentially expressed proteins (DEPs) were identified by Perseus software (*p* < 0.05) (Figure 4A). Compared with WT cattle, there were 25 down-regulated proteins (FC < 0.5) and 50 up-regulated proteins (FC > 2) in MT cattle. Both cluster analysis and principal component analysis showed significant differences between MT cattle and WT cattle.

**Figure 5:**
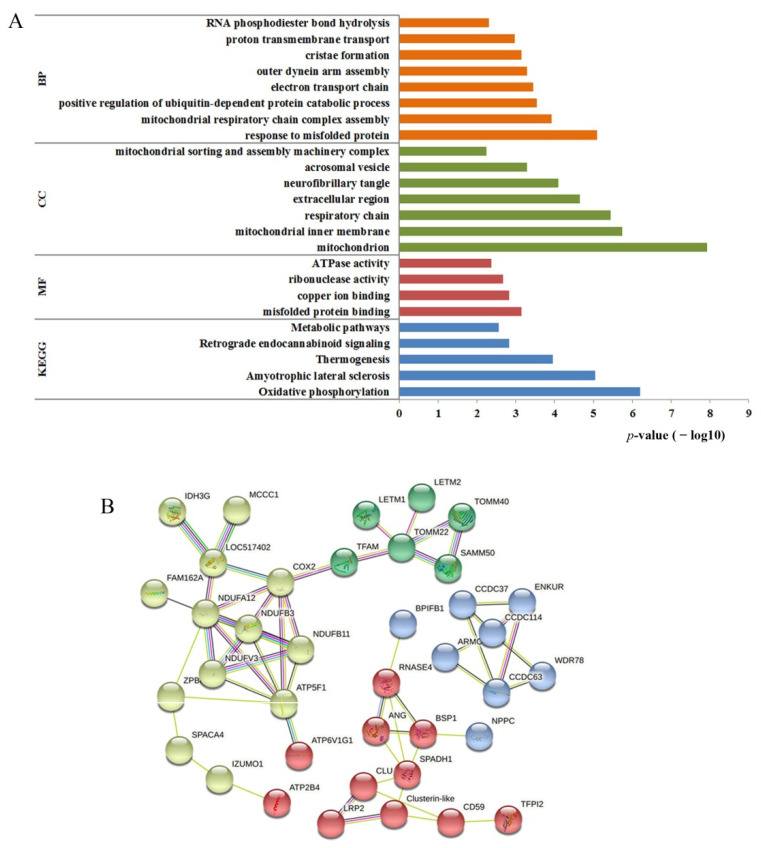
Functional analysis of differentially expressed proteins. (**A**) The results of GO and KEGG; (**B**) protein interaction network using STRING.

The biological functions of DEPs were analyzed by gene ontology (GO) annotations. Annotation of biological processes (BPs) were mainly involved in response to misfolded protein, mitochondrial respiratory chain complex, positive regulation of ubiquitin-dependent protein catabolic process, electron transport chain, outer dynein arm assembly, cristae formation, proton transmembrane transport, and RNA phosphodiester hydroxyapatite (Figure 5A). The enriched terms in cellular components (CCs) were mitochondrion, mitochondrial inner membrane, respiratory chain, extracellular region, fibrotic tangle, acrosomal vesicle, mitochondrial sorting, and assembly machinery complex. The main rich entries of molecular functions (MFs) included misfolded protein binding, copper ion binding, ribonuclease activity, and ATPase activity. The KEGG pathway-enrichment analysis showed that the DEPs were involved in oxidative phosphorylation, amyotrophic lateral sclerosis, thermogenesis, retrograde endocannabinoid signaling, and metabolic pathways.

The interaction network of the 75 DEPs was analyzed by String software. Among them, 37 proteins had no interaction, which is not shown in Figure 5B. The remaining 38 proteins formed three small interaction networks (Figure 5B). The regulatory networks formed by yellow- and green-colored proteins were mainly associated with mitochondrial function. The yellow-colored proteins were mainly related to energy metabolism, such as NADH dehydrogenase family proteins and ATP synthase family proteins. The green-colored proteins were related to the mitochondrion, such as the mitochondrial import protein, LETM1, and EF-hand domain-containing protein 1, which mediates proton-dependent calcium efflux from mitochondrion. The blue-colored proteins were mainly structural proteins of sperm, such as cilia- and flagella-associated proteins and coiled-coil domain-containing proteins. The red-colored proteins were regulatory proteins, such as spermadhesin-1 precursor and clusterin protein, which regulate spermatogenesis and maturation.

The biological functions of 75 DEPs were analyzed in detail using Uniprot database. The results showed that 20 proteins were associated with mitochondrion, and 9 proteins were related to sperm motility (Table 8). Among the 20 mitochondria-related proteins, only ATP5F1 was significantly down-regulated, while the other 19 proteins were significant up-regulated in MT cattle. The protein expressions of nine motility-related proteins were all up-regulated in MT cattle. At the same time, we found the ANG1, a binding regulator of copper ions, was down-regulated in MT cattle, which was consistent with the copper decrease in seminal plasma. The KCNU1 was related to the activation of potassium-calcium channels, which was up-regulated in MT cattle and was consistent with the increase of potassium in seminal plasma.

### 3.7. The Fertilization Ability of Cryopreserved Semen of MSTN Gene-Edited Cattle

The cryopreservation semen of MT bulls was used for artificial insemination. Of the 97 inseminated cows, 65 were pregnant, with a pregnancy rate of 67%; 53 calves were born, and the calving rate was 81.5%, which indicated that the cryopreserved MT cattle semen is fertile and can be used in beef cattle production.

## 4. Discussion

### 4.1. Effects of MSTN Mutation on Bovine Growth Traits

At present, there are three kinds of gene-editing tools: (1) zinc finger nuclease (ZFN); (2) transcription activator-like effector (TALEN); and (3) clustered, regularly interspaced short palindromic repeats (CRISPR)/Cas (CRISPR associated proteins). In 2002, ZFN technology was first applied to knock out the target gene and then apply it in animal genome editing [22,23]. However, due to high cost and low efficiency, ZFN has not become a widely used editing tool. In 2011, TALEN was successfully applied to gene editing [24,25,26]. Compared with ZFN, TALEN is more efficient, but the construction of the target module of TALEN is still complex. Since 2013, the initial application of the CRISPR/Cas9 technology in mammals, the reports of this system for genomic editing have skyrocketed [27,28,29]. In this study, we obtained eight gene-edited cattle at one time through CRISPR/Cas9, and these MSTN-mutation cattle improved meat production performance on the one hand and maintained the characteristics of the original strain on the other hand.

Studies have shown that with Belgian Blue and Piedmontese as terminal crossbred bulls, muscle mass of F1 generation can be increased by 20–25% [30,31,32]. Casas et al. showed that MSTN hybrid cattle have larger sectional area of rib-eye meat, thinner back fat, higher slaughter rate, and lower fat content than the common cattle [33]. Coopman et al. reported that the average body weight of Belgian Blue bulls was 98 kg and 242 kg at 3 and 7 months of age, respectively, and that of common cows was about 96 kg and 189 kg, respectively, at the same month of age [32]. Genicot et al. reported the body weight of Belgian Blue bulls was 242 kg at 6 months of age [30]. Cundiff et al. reported the average body weight of Piedmontese cattle was 220 kg at 7 months of age [34]. According to the production performance parameters of Chinese Simmental cattle, the weaning weights were 210 kg and 190 kg for bull and cow, respectively [35]. The average weight of 1050 Luxi cattle was 125 kg at 6 months of age. The body weight of the F1 generation hybrid of Qinghai indigenous Yellow cattle and Piedmontese were 80.7 kg, 140.5 kg, and 270.0 kg for bulls at 3, 6, and 12 months of age, respectively, and for cows, the weights were 77.3 kg, 132.1 kg, and 249.6 kg, respectively [33]. The results in this study showed no significant difference in body length, hip height, shoulder width, and hip width between MT cattle and WT cattle before 6 months of age. At 9 months of age, the growth traits of MT cattle were generally better than WT cattle, especially for the body weight, shoulder width, and hip width, and the difference was significant at 12 months of age. These results indicate that the *MSTN* gene-edited cattle have excellent growth traits.

### 4.2. Effects of MSTN Mutation on Physiological and Biochemical Indexes of Cattle

Serum physiological and biochemical parameters can reflect the physiological and metabolic status of entire body of animals; meanwhile, they are an important reference index for studying the mechanism of toxicology and pathology. In this study, the blood biochemical and physiological parameters of MT and WT cattle at 9, 12, 15, and 18 months of age were analyzed, respectively. As shown in Table 3, there were no significant differences between MT and WT cattle in the indexes of red blood cell count, hematocrit, hemoglobin, platelet count, platelet hematocrit, lymphocyte ratio, and median cell count. These results indicate MT cattle have similar physiological parameters as WT cattle.

In the measured 18 biochemical parameters in this study, 11 of them had no statistical differences between MT and WT cattle, but the other 7 parameters, namely glucose (GLU), high-density lipoprotein (HDL), low-density lipoprotein (LDL), alpha-amylase (PAMY), lactic acid (LA), urea (BUN), and lipase (LIP), had significant differences (Table 4). The glucose level of MT cattle was significantly lower than that of WT cattle, while the lipase, urea, lactic acid, and amylase activity of MT cattle were significantly higher, which suggests that mutation of the *MSTN* gene changed the metabolism of glucose, lipids, and proteins. Our most recent reports showed that *MSTN* gene mutation in cattle induced complex and elaborate mechanisms to skeletal muscle, heart muscle, stomach microbe, and bile acid metabolism [19,36,37,38]. The biological significances of the changes in biochemical parameters need further investigation.

### 4.3. Effects of MSTN Mutation on Bovine Semen and Spermatozoa

Semen characteristics of *MSTN*-gene-edited bulls, including semen volume, sperm density, fresh sperm motility, and frozen-thawed sperm motility, have no significant difference from that of WT bulls. MT bull semen is fertile and can be used for beef cattle production. The trace elements in semen are crucial to sperm function, and the lack of them may be a significant factor for impaired spermatogenesis, poor sperm quality, and poor fertility [39,40]. The trace elements of seminal plasma were analyzed by ICP-MS and showed that the concentrations of K, Hg, and Sr in MT bulls were higher than that in WT bulls, while V, Cu, Mn, Sn, Sb, and Co were significant lower. In humans, Hamad et al. showed that infertile patients had a significantly low concentration of seminal K compared to fertile males, while the Cu level appeared to be decreased in fertile males compared to infertile males [41].

Motility properties of sperm are significantly weighted among various functional parameters of sperm, such as tail vibrations that enable sperm to move forward after ejaculation [42]. In bulls, major morphological sperm abnormalities are associated with reduced fertility, and morphological assessments have been used to provide an indication of an individual’s potential fertility [43]. Therefore, in the present study, the sperm motility-related parameters, plasma membrane integrity, acrosome integrity, and mitochondrial membrane integrity were examined to investigate the effects of MSTN mutation on sperm. We found that WOB (wobble) and BCF (beat cross-frequency) of MT cattle spermatozoa were significantly higher than that of WT, while the mitochondrial membrane integrity was significant lower.

Genetic studies have demonstrated that MSTN deficiency leads to muscle hypertrophy due to a combination of increased myofiber numbers and increased myofiber sizes in multiple species, including humans, sheep, dogs, and cattle, without causing severe adverse consequences [38,44]. Mosher et al. reported that disruption of *MSTN* gene function increased overall athletic performance in individuals [45]. Therefore, the sperm motility of MT bulls might be enhanced, and the flagellum fiber of sperm might be increased. Our previous reports have shown that the glycolysis, glycogen metabolism, and fatty acid β-oxidation was significantly changed in MT cattle [37,46,47,48]. Meanwhile, various effects of *MSTN* gene mutation on cattle and the related molecule mechanism have been investigated, including bile acid metabolism [19], enhanced glycolysis in the heart [37], and microbial community of stomach [36]. The energy metabolism was changed in MT cattle, so the sperm mitochondria should be also affected.

In order to verify our hypothesis for the effect of *MSTN* gene editing on cattle spermatozoa, FLQ proteomics technique was used to identify the DEPs of cattle spermatozoa between MT and WT. A total of 2279 proteins were identified, with 75 DEPs, including 50 up-regulated proteins and 25 down-regulated proteins. Biological functions of DEPs were mainly related to mitochondria and fibrous sheath. Among the 75 DEPs, there were 20 proteins were associated with mitochondrion, and 9 proteins were related to sperm motility (Table 8).

The nine motility-related proteins were all up-regulated in MT cattle, including three CCDC family proteins (CCDC63, CCDC114 and CFAP100, also known as CCDC37), two dynein proteins (ARMC4 and WDR78), TSSK4, ENKUR, FSCB, and TUBB4A. Sperm flagella typically comprise a “9 + 2” axonemal arrangement and a number of multi-protein complexes, including outer dense fiber (ODF), fibrous sheath (FS), nexin-dynein regulatory complex (NDRC), calmodulin-and spoke-associated complex (CSC), and dynein arms (DAs) [49]. *ARMC4* and *WDR78* belong to the ODF. Mutations in *ARMC4* lead to low expression of ARMC4 protein in sperm flagella, which might cause male infertility with primary ciliary dyskinesia in humans, mice, and zebrafish and impacts the ODF ultrastructure of mutated spermatozoa [50]. Functional defects for the WDR78 cause complete inability to assemble the axoneme in vertebrates and results in infertility [49]. Coiled-coil domain-containing proteins play important roles in cilia- and flagella-associated functions, and the proven genes are *CCDC9*, *CCDC39*, *CCDC42*, *CCDC63*, *CCDC113*, *CCDC135*, *CCDC147*, *CCDC151*, *CCDC181* and *CCDC189* [51]. The testis-enriched *CCDC42* performs essential functions in connecting the sperm flagella and sperm head. *CCDC42*-KO male mice display sperm flagellar defects and are sterile. *CCDC63*-KO rendered the mice infertile, with the spermatozoa having shortened flagella [52].

The testis-specific serine/threonine kinase (TSSK) has been demonstrated to play essential roles in spermatogenesis, such as *TSSK1b*, *TSSK2*, and *TSSK4* encode kinases regulating sperm motility and fertility [53]. The gene *TSSK4* has proven to be essential for maintaining the structural integrity of sperm flagellum, and *TSSK4*-KO mice exhibit an impaired sperm structure and reduced sperm motility, which affects fertility [51], and up-regulated TSSK4 can restore fertility of male mice [54]. One novel protein kinase A-phosphorylated calcium-binding protein, FSCB, has a temporal appearance during spermatogenesis and is located at the cortex of the fibrous sheath, involved in late steps of fibrous sheath biogenesis [55]. Protein FSCB is capable of being phosphorylated and binding to calcium, which enhances the spermatozoa flagellar movement and spermatozoa capacitation and activation [56]. The fibrous sheath affects flagellar beating via the scaffolding of signaling pathways necessary for motility [57].

Protein ENKUR, known as *Enkurin*, a novel TRPC channel interacting protein, localizes on the head and flagellum and regulates flagellar motility [58]. Surprisingly, the CCDC, WDR, and CFAP protein families are interrelated. The CFAP protein family, encoding for WD repeat domains (WDR) containing proteins, always contains coiled-coil domains, which are likely to govern sperm flagellum assembly, organization, and beating [59]. In this study, the increase of WOB and BCF in MT bulls sperm reflected an enhancement of flagellum wobble and beating, which may be due to the up-regulation of motility-related proteins.

The increase of sperm wobble and beating requires more energy supply. Our previous studies proved that the glycolysis and glycogen metabolism was significantly enhanced in MT cattle, which provided more energy for muscle growth and athletic activity [47,48]. For the sperm, the main energy supply structure is mitochondria. Nineteen mitochondrial-related proteins were significantly up-regulated in the present sperm proteomic experiment, which were mainly involved in oxidative phosphorylation and thermogenesis pathways. The enhancement of sperm energy metabolism, on the one hand, provides enough energy for sperm wobble and beating; on the other hand, hyper-activation of mitochondrial metabolic induces more severe stress response of sperm during freezing and thawing, resulting in aggravation of mitochondrial damage, which coincides with the significant decline of mitochondrial membrane integrity of MT spermatozoa. According to the characteristics of sperm, MT sperm shows higher activity compared to WT, but the cryoinjury of MT sperm seems to be more serious. Therefore, the quality of frozen spermatozoa of MT bulls can be further improved by optimizing the composition of cryodiluent solution, such as adding antioxidants. This will be further studied in our future research work.

## 5. Conclusions

Bulls edited with myostatin gene by CRISPR/Cas9 technique show significant improvement in growth traits, particularly hip widths and hindquarters. Most physiological and biochemical parameters of MSTN mutant bulls are similar to that of wild-type bulls, but several indexes reflect the effect of the gene mutation. The wobble and beating of MT bull spermatozoa are significantly higher than that of WT bulls, which implies that the sperm have higher motility and possible improved fertility. Sperm FLQ proteomic analysis further revealed that 9 motility-related proteins and 19 mitochondrial-related proteins were significantly up-regulated in MT spermatozoa, respectively, which may play a role to govern sperm flagellum assembly, organization, and beating and provide sufficient energy for sperm motility. In conclusion, the *MSTN* gene-edited bulls have improved growth traits and biochemical indexes and have normal fertility and can be used for beef cattle production.

## Figures and Tables

**Table 1:** Ingredients and nutrients of the commercially manufactured feeds.

Items	Value (% of Dry Matter)	
	Special Feed for Calves	Special Feed for Bulls
Crude protein	>18.0	>16.0
Crude ash	<14.0	<12.0
Crude fiber	<6.0	<9.0
Calcium	0.6–1.2	0.5–1.8
Phosphorus	>0.4	>0.4
NaCl	0.5–1.0	0.8–1.5
Lysine	>0.7	>0.6

**Table 2:** Cattle body weight at different month age.

Months of Age	MT (kg)	WT (kg)	Difference Value (kg)	Increase Rate (%)
0	33.20 ± 1.23	30.67 ± 2.85	2.53	8.25
3 *	111.40 ± 6.45	99.05 ± 7.46	12.35	12.47
6 *	184.71 ± 12.16	167.20 ± 16.32	17.51	10.47
9 *	252.82 ± 15.05	213.37 ± 20.05	39.45	18.49
12 *	335.67 ± 16.27	280.13 ± 19.75	55.54	19.83
15 *	412.76 ± 19.56	328.56 ± 24.05	84.20	25.63
18 *	482.51 ± 18.94	402.25 ± 24.58	80.26	19.95
24 *	593.59 ± 21.64	485.73 ± 28.17	107.86	22.21

Note: The difference value represents the weight of the edited cow minus the weight of the regular cattle. The increase rate is the difference divided by the weight of the regular cattle multiplied by 100%. * stands for *p* < 0.05.

**Table 3:** Routine blood tests of cattle at different months of age.

Index	MT				WT			
	9	12	15	18	9	12	15	18
LYM%	6.01 ± 1.38	6.81 ± 2.76	5.53 ± 1.60	5.12 ± 0.13	6.09 ± 3.43	6.87 ± 2.34	5.47 ± 2.19	5.53 ± 0.60
LYM	0.38 ± 0.14	0.43 ± 0.43	0.34 ± 0.13	0.38 ± 0.09	0.82 ± 0.25	0.46 ± 0.61	0.41 ± 0.22	0.37 ± 0.32
NEUT	1.09 ± 0.47	0.98 ± 0.43	0.69 ± 0.12	0.56 ± 0.13	1.10 ± 1.35	0.90 ± 0.81	0.66 ± 0.44	0.67 ± 0.61
RBC	9.10 ± 1.10	9.18 ± 0.080	9.01 ± 0.33	8.88 ± 0.34	9.20 ± 0.19	9.01 ± 0.21	8.87 ± 1.23	87.77 ± 0.32
MCV	3504 ± 254.21	3511 ± 345.30	3489 ± 309.30	3501 ± 280.34	3434 ± 234.01	3419 ± 276.07	3402 ± 151.67	3487 ± 154.32
PLT	286.31 ± 63.76	266.51 ± 72.52	254.12 ± 82.03	248.81 ± 81.09	278.39 ± 167.12	268.43 ± 30.01	243.42 ± 65.45	271.13 ± 51.22
PCT	0.23 ± 0.16	0.22 ± 0.13	0.22 ± 0.09	0.19 ± 0.14	0.22 ± 0.78	0.22 ± 0.56	0.21 ± 0.11	0.21 ± 0.12
MPV	6.76 ± 0.34	6.77 ± 0.70	6.78 ± 0.08	6.76 ± 0.50	7.01 ± 1.09	7.02 ± 0.04	7.02 ± 0.32	7.00 ± 0.21
BA	0.11 ± 0.03	0.10 ± 0.05	0.10 ± 0.05	0.11 ± 0.03	0.11 ± 0.07	0.10 ± 0.07	0.10 ± 0.02	0.10 ± 0.03
BA%	1.02 ± 0.17	1.06 ± 0.09	0.99 ± 0.54	1.05 ± 0.35	1.07 ± 0.32	0.99 ± 0.87	0.98 ± 0.90	1.06 ± 0.11
AL%	0.88 ± 0.12	0.87 ± 0.49	0.79 ± 0.80	0.78 ± 0.10	1.04 ± 0.50	1.06 ± 0.21	0.88 ± 0.66	0.67 ± 0.16
AL	0.07 ± 0.04	0.07 ± 0.01	0.06 ± 0.04	0.06 ± 0.02	0.08 ± 0.04	0.08 ± 0.02	0.07 ± 0.08	0.07 ± 0.05
HGB	118.60 ± 10.43	116.44 ± 13.13	118.9 ± 10.33	116.4 ± 9.92	117.60 ± 9.76	117.43 ± 12.41	116.78 ± 8.48	116.35 ± 2.11

**Table 4:** Physiological and biochemical indexes of cattle at different months of age.

Index	MT				WT			
	9	12	15	18	9	12	15	18
GLU *	3.87 ± 0.77	3.82 ± 1.04	3.84 ± 1.02	3.81 ± 0.70	4.47 ± 0.24	4.41 ± 0.33	4.39 ± 0.67	4.68 ± 0.27
ASP	58.64 ± 3.23	57.77 ± 12.54	60.65 ± 12.70	60.71 ± 8.24	57.83 ± 7.56	58.03 ± 7.21	59.06 ± 5.53	58.75 ± 4.59
ALT	24.86 ± 7.72	24.93 ± 3.43	25.01 ± 5.43	25.42 ± 5.18	25.12 ± 6.62	25.13 ± 3.44	24.31 ± 5.27	25.05 ± 4.01
TP	66.68 ± 1.66	65.79 ± 7.34	65.34 ± 7.41	66.07 ± 5.78	65.09 ± 8.34	65.77 ± 6.43	65.33 ± 5.34	66.08 ± 3.78
ALB	36.38 ± 2.35	35.85 ± 7.31	36.01 ± 7.43	36.07 ± 5.21	36 ± 3.07	36.17 ± 3.12	35.90 ± 7.84	36.45 ± 1.11
CK	193 ± 67.71	189 ± 54.32	192.70 ± 56.45	190.11 ± 52.43	189.87 ± 59.03	192.34 ± 61.01	187.47 ± 28.42	194.31 ± 16.41
HDL *	3.03 ± 0.76	3.01 ± 0.87	2.97 ± 0.69	3.08 ± 0.36	2.11 ± 0.43	2.24 ± 0.87	2.26 ± 0.52	2.09 ± 0.89
LDL *	1.55 ± 0.64	1.58 ± 0.43	1.54 ± 0.21	1.66 ± 0.31	0.79 ± 0.13	0.85 ± 0.56	0.85 ± 0.29	0.99 ± 0.65
PAMY **	26.61 ± 5.12	26.44 ± 5.12	34.95 ± 6.67	35.19 ± 6.31	25.91 ± 7.12	23.21 ± 3.54	21.06 ± 7.45	22.27 ± 7.56
CREA	138.21 ± 40.01	139.21 ± 18.29	139.43 ± 25.33	138.92 ± 17.21	138.12 ± 23.30	137.54 ± 32.61	135.38 ± 17.54	138.22 ± 7.01
LDH	1108.34 ± 125.43	1108.76 ± 165.22	1110.32 ± 134.42	1103.87 ± 166.11	1110.16 ± 232.02	1113 ± 137.32	1094.54 ± 165.21	1096.24 ± 55.13
AB	22.17 ± 2.54	22.32 ± 4.77	22.88 ± 3.76	23.90 ± 2.10	21.82 ± 4.12	22.01 ± 3.05	21.25 ± 3.31	22.81 ± 1.89
CHE	143.57 ± 22.34	144.09 ± 24.32	143.21 ± 43.55	145.87 ± 22.42	144.15 ± 28.77	143.54 ± 18.04	144.33 ± 18.06	144.70 ± 35.23
TC	3.02 ± 0.78	3.03 ± 1.01	3.11 ± 1.04	3.07 ± 0.76	3.01 ± 0.12	3.01 ± 0.54	3.03 ± 0.87	3.06 ± 0.15
LA *	4.38 ± 2.66	3.97 ± 3.34	3.87 ± 2.79	3.96 ± 1.41	2.47 ± 0.70	2.44 ± 0.78	2.41 ± 0.43	2.35 ± 0.81
TG	0.31 ± 0.21	0.29 ± 0.12	0.30 ± 0.09	0.31 ± 0.21	0.31 ± 0.02	0.29 ± 0.09	0.29 ± 0.02	0.30 ± 0.11
BUN **	2.53 ± 3.21	2.55 ± 1.87	2.65 ± 1.34	2.61 ± 0.36	1.30 ± 0.60	1.32 ± 0.43	1.28 ± 0.49	1.31 ± 0.54
LIP **	15.72 ± 3.16	15.81 ± 5.06	15.59 ± 6.10	16.49 ± 9.10	11.54 ± 5.32	12.10 ± 4.14	12.06 ± 5.02	11.95 ± 6.21

Note: * stands for *p* < 0.05, ** stands for *p* < 0.01.

**Table 5:** Semen characteristics of cattle.

Bulls	Semen Volume (ml)	Sperm Density (10^6^/mL)	Fresh Sperm Motility (%)	Frozen Sperm Motility (%)
MT1	5.6 ± 2.6	1538 ± 504	78.9 ± 9.7	49.1 ± 13.7
MT2	5.1 ± 1.9	1344 ± 616	75.1 ± 14.2	37.6 ± 15.9
MT3	4.1 ± 1.8	1258 ± 346	84.0 ± 4.8	62.1 ± 8.8
MT4	3.9 ± 0.4	1024 ± 247	63.3 ± 8.4	18.8 ± 11.6
MT5	5.5 ± 2.6	1242 ± 564	72.9 ± 10.8	42.3 ± 10.0
MT6	4.8 ± 1.7	1109 ± 462	69.4 ± 9.1	34.9 ± 10.8
Average of MT	4.8 ± 0.7	1252 ± 180	73.9 ± 7.2	40.8 ± 14.5
WT1	5.7 ± 1.1	1477 ± 612	74.6 ± 9.0	45.7 ± 9.2
WT2	5.1 ± 1.2	1462 ± 632	78.3 ± 7.9	56.2 ± 5.8
WT3	5.9 ± 1.1	2118 ± 695	81.9 ± 6.2	65.6 ± 9.8
Average of WT	5.6 ± 0.3	1686 ± 306	78.3 ± 3.0	55.8 ± 8.1
*p*-value	0.09	0.17	0.27	0.12

**Table 6:** Trace elements in bovine seminal plasma.

Elements	MT1	MT2	MT3	MT4	MT5	MT6	WT1	WT2	WT3
Na (mg/L)	372.9	308.2	169.3	276.4	298.7	346.2	281.0	407.3	446.9
K * (mg/L)	202.7	65.8	395.9	246.8	223.0	89.9	105.4	75.3	79.3
Ca (mg/L)	381.1	249.2	212.2	164.1	235.0	224.2	259.2	328.6	229.8
Mg (mg/L)	49.0	45.4	46.8	26.6	39.2	43.2	26.4	77.4	46.0
Fe (mg/L)	6.8	4.3	3.0	2.4	3.3	3.1	3.9	7.2	3.9
Zn (mg/L)	6.3	4.4	3.4	2.1	3.1	2.6	2.2	8.6	4.3
Se (μg/L)	460.5	450.6	418.6	249.1	363.9	502.4	ND	985.2	151.9
Cr (μg/L)	433.8	386.5	271.4	172.9	274.9	306.1	85.6	420.5	174.7
Ti (μg/L)	170.1	180.6	114.1	68.3	79.2	83.1	136.8	159.3	121.1
V ** (μg/L)	135.3	121.4	88.1	59.4	88.8	98.6	442.6	515.0	385.2
Al (μg/L)	125.8	146.9	51.3	ND	ND	ND	1.5	59.3	17.2
Hg ** (μg/L)	112.2	88.2	80.9	82.6	82.3	81.4	0.7	1.7	0.9
Sr ** (μg/L)	99.1	86.8	88.0	114.1	102.3	78.2	33.0	38.0	25.4
Ba (μg/L)	52.3	73.8	32.1	90.9	40.7	44.5	105.5	241.0	101.6
Cu * (μg/L)	48.5	102.1	54.1	27.2	40.1	68.3	360.2	562.9	397.7
Mn * (μg/L)	22.3	19.7	16.2	99.1	8.6	7.0	70.2	172.1	115.5
Ni (μg/L)	37.1	26.0	18.9	15.0	20.5	19.8	28.1	14.7	9.6
Pb (μg/L)	11.3	13.7	9.5	9.9	10.8	9.4	1.3	33.1	7.4
Sn * (μg/L)	9.9	10.0	7.3	7.8	7.8	7.3	36.0	93.6	76.1
Sb * (μg/L)	8.1	8.0	4.9	0.1	ND	ND	80.8	175.2	84.7
Co ** (μg/L)	2.3	0.7	0.1	0.2	ND	0.2	9.6	15.0	12.6

Note: ND indicates no detection. * stands for *p* < 0.05, ** stands for *p* < 0.01.

**Table 7:** Sperm motility-related parameters of MSTN-edited bovine.

Indexes	MT1	MT2	MT3	MT4	MT5	MT6	WT1	WT2	WT3
VCL (mm/s)	77.6	61.7	79.9	63.8	73.0	51.4	66.2	52.7	64.0
VSL (mm/s)	37.2	24.7	29.6	27.4	27.7	20.8	35.1	25.2	26.0
VAP (mm/s)	48.0	35.0	47.0	38.2	39.9	29.7	44.5	32.3	36.9
LIN (%)	43.5	33.6	35.2	38.3	33.9	33.8	44.0	38.2	33.4
STR (%)	67.8	57.2	58.2	61.7	58.6	56.6	65.2	61.5	56.9
WOB * (%)	59.7	57.0	57.9	57.8	63.5	64.5	57.9	55.2	54.3
ALH (um)	3.2	2.9	3.4	2.8	3.3	2.4	2.6	2.3	2.9
BCF * (Hz)	8.4	6.8	7.7	6.6	6.3	6.3	6.4	6.3	5.8

Note: * stands for *p* < 0.05.

**Table 8:** Partly DEPs of cattle spermatozoa between MT and WT.

Accession.	Description	*p*-Value	FC	Regulated
Q58D96	Isocitrate dehydrogenase [NAD] subunit, mitochondrial GN = IDH3G	0.041	5.28	UP
Q58DG1	UPF0160 protein MYG1, mitochondrial GN = MYG1	0.005	4.12	UP
P13619	ATP synthase F(0) complex subunit B1, mitochondrial GN = ATP5F1	0.032	0.08	DOWN
F1N3R4	Mitochondrial proton/calcium exchanger protein GN = LETM1	0.012	5.72	UP
E1BA21	Transcription factor A, mitochondrial GN = TFAM	0.042	13.64	UP
E2GEZ1	Translocase of outer mitochondrial membrane 22 GN = Tom22	0.017	2.91	UP
E2GEZ2	Translocase of outer mitochondrial membrane 40A GN = Tom40A	0.023	4.78	UP
Q58D49	Cob(I)yrinic acid a,c-diamide adenosyltransferase, mitochondrial GN = MMAB	0.042	4.12	UP
Q8HXG5	NADH dehydrogenase [ubiquinone] 1 beta subcomplex subunit 11, mitochondrial GN = NDUFB11	0.019	8.17	UP
P25712	NADH dehydrogenase [ubiquinone] flavoprotein 3, mitochondrial GN = NDUFV3	0.012	9.93	UP
Q02365	NADH dehydrogenase [ubiquinone] 1 beta subcomplex subunit 3 GN=NDUFB3	0.032	2.74	UP
E1B9Z2	Leucine zipper and EF-hand containing transmembrane protein 2 GN = LETM2	0.014	7.14	UP
O97725	NADH dehydrogenase [ubiquinone] 1 alpha subcomplex subunit 12 GN = NDUFA12	0.023	7.00	UP
D3K0R6	Plasma membrane calcium-transporting ATPase 4 GN = ATP2B4	0.029	3.42	UP
Q2HJ55	Sorting and assembly machinery component 50 homolog GN = SAMM50	0.038	12.07	UP
P68530	Cytochrome c oxidase subunit 2 GN = MT-CO2	0.016	9.01	UP
A5PKG4	Cysteine desulfurase, mitochondrial GN = NFS1	0.015	3.98	UP
F1MEM9	Tetratricopeptide repeat domain 19 GN = TTC19	0.044	6.31	UP
Q2NKR7	Protein FAM162A GN = FAM162A	0.001	8.70	UP
E1BGC1	Methylcrotonoyl-CoA carboxylase 1 GN = MCCC1	0.019	7.12	UP
Q3ZBU7	Tubulin beta-4A chain GN = TUBB4A	0.044	6.27	UP
E1B8W3	Outer dynein arm docking complex subunit 2 GN = ARMC4	0.043	3.71	UP
Q2T9N0	Fibrous sheath CABYR-binding protein GN = FSCB	0.020	4.04	UP
Q2T9W3	Coiled-coil domain-containing protein 63 GN = CCDC63	0.032	10.17	UP
F1N2N9	Coiled-coil domain-containing 114 GN = CCDC114	0.025	3.38	UP
E1B836	Enkurin, TRPC channel interacting protein GN = ENKUR	0.035	3.28	UP
E1B9S6	Dynein axonemal intermediate chain 4 GN = WDR78	0.040	2.24	UP
F6RLA2	Testis specific serine kinase 4 GN = TSSK4	0.002	7.74	UP
F6QYE2	Cilia and flagella associated protein 100 GN = CFAP100	0.004	7.37	UP
P10152	Angiogenin-1 GN = ANG1	0.006	0.01	DOWN
C6KH61	Potassium calcium-activated channel subfamily U member 1 GN = KCNU1	0.005	4.23	UP

Note: The FC represents the fold changes of protein expression between MT and WT cattle. The mitochondria-related proteins are shown with white background. The sperm motility-related proteins are shown with green background. The ions binding-related proteins are shown with blue background.

## Data Availability

The data presented in this study are available on request from the corresponding author.
